# Midwives' challenges in the management of postpartum haemorrhage at rural PHC facilities of Limpopo province, South Africa: an explorative study

**DOI:** 10.4314/ahs.v21i1.40

**Published:** 2021-03

**Authors:** Thifhelimbilu Irene Ramavhoya, Maria Sonto Maputle, Rachel Tsakani Lebese, Lufuno Makhado

**Affiliations:** 1 Department of Nursing Sciences, Faculty of Health Sciences, University of Pretoria, South Africa; 2 Department of Advanced Nursing Science, University of Venda, Private Bag X5050, Thohoyandou; 3 Research office, School of Health Sciences, University of Venda, South Africa

**Keywords:** Midwifery in South Africa, implementation of maternal guidelines, postpartum haemorrhage, maternal mortality

## Abstract

**Background:**

Postpartum haemorrhage is one of the causes of the rise in maternal mortality. Midwives' experiences related to postpartum haemorrhage (PPH) management remain unexplored, especially in Limpopo. The purpose of the study was to explore the challenges experienced by midwives in the management of women with PPH.

**Methods:**

Qualitative research was conducted to explore the challenges experienced by midwives in the management of women with PPH. Midwives were sampled purposefully. Unstructured interviews were conducted on 18 midwives working at primary health care facilities. Data were analysed after data saturation.

**Results:**

After data analysis, one theme emerged “challenges experienced by midwives managing women with PPH” and five subthemes, including: “difficulty experienced resulting in feelings of frustrations and confusion and lack of time and shortage of human resource inhibits guidelines consultation”.

**Conclusion:**

The study findings revealed that midwives experienced difficulty when managing women with postpartum haemorrhage. For successful implementation of maternal health care guidelines, midwives should be capacitated through training, supported and supervised in order to execute PPH management with ease.

## Introduction

Post-Partum Haemorrhage (PPH) remains the leading cause of deaths amongst women all over the world. PPH accounts for 30% of maternal death which equal 86 000 deaths per year or ten deaths per hour.^1^ One of the complications of the third and fourth stage of labour was discovered to be PPH.^2^ PPH is preventable however, with 275 000 births in sub-Saharan Africa, 1.2% of women suffer from PPH. It was indicated that sub-standard care, poor management skills, lack of knowledge and delay in transferring women to the next level were amongst the causes of increased maternal mortality rate.^3^ The rate of maternal mortality caused by preventable conditions was found to be high in Limpopo Province. The Province was ranked number three on the high maternal mortality rate (MMR) as indicated in Saving Mothers report 2011–2013.^4^ Obstetric haemorrhage which encompasses PPH has ranked number two and accounts to 38.2% of maternal deaths reported at PHC facilities in South Africa.^4^

Regardless of the use of maternal health guidelines during the provision of maternal health care services, pregnant women and mothers are still dying from obstetric haemorrhage. The maternity care guidelines outline the important aspects that need to be followed by midwives with the aim of saving the lives of mothers if they are implemented correctly and timeously. In order for midwives to be able to implement the maternity care guidelines; they should have acquired the necessary knowledge and skills to manage PPH. When the midwife diagnoses the women with PPH, it is expected for her to call for assistance, rub the fundus and expel clots, administer two IV lines using wide IV cannula in order to replace blood loss.^5^ Midwives must identify the cause of PPH and treat the women accordingly. If the cause of PPH is a vaginal tear, to be repaired immediately, if it is retained products of the placenta, they must also be removed.

The World Health Organisation (WHO) recommended that Oxytocin alone is effective in the management of PPH instead of adding syntometrine or ergometrine as a first-line drug as this is the second-line drugs.^5^ Oxytocin 20mg must be added in one of the drips, 1000mls of Ringers lactate at 125ml/hour to stimulate uterine contractions.^6^ Apart from the use of uterotonics, a new method was discovered in the management of PPH called the butterfly where compression of the uterus was seen as easy.^7^ The women must be transferred immediately to the hospital. If resuscitation through medical interventions is not effective, surgical intervention may be required in order to save the lives of women though it will be done at the hospital level.^8^

Regardless of the management indicated above, midwives are faced with challenges that enabled them to can manage the women effectively hence, these challenges contribute to a high maternal mortality rate. 9 Inadequate staffing places workload to midwife on duty, hence the midwife will be in a hurry to can see all the patients for that day. Apart from attending to maternal health care services which entail attending to pregnant women following basic ante-natal care, delivering women when in labour and post-natal care, comprehensive primary health care services are offered. Minor ailments are treated, and chronic conditions are attended to by midwives. Hence most of the time, this care is offered by one midwife, assisted by an enrolled or an assistant nurse. Pregnant women failed to disclose their previous history of PPH due to fear of being transferred to a high-risk clinic in the hospital which involves travelling costs. Shortage of material resources such as ambulances that is not always there when in need of transferring the women to the next level.^9^, ^10^ Poor communication between the staff where those who attended workshops failed to disseminate information in time hence poor implementation of the guideline when managing the women with PPH.^9^,^10^ Though continuous training and staff development was done through Essential Management of Obstetric Emergencies (ESMOE), maternal morbidity and mortality rate is still high. MMR caused by obstetric haemorrhage was at 33.59/100 000 live birth, making Limpopo the leading province from all the provinces.^4^ Hence the two selected districts had MMR of 288/100 000 live birth caused by preventable conditions such as PPH. The need to conduct this study aroused from this background and prompted the researchers to explore the challenges experienced by midwives in the management of women with PPH

## Methods and Design

The qualitative descriptive exploratory research design was used to describe the challenges experienced by midwives in the management of women with Post-Partum Haemorrhage (PPH). The reason for choosing this method was to obtain more information on how midwives managed women who experienced PPH. This study was conducted from the rural Primary Health Care (PHC) facilities of the two-selected district at Limpopo province. PPH was ranked number two amongst other preventable conditions responsible for maternal mortality rate in South Africa hence, the two districts had a high maternal mortality rate of 235 per 100 000 live birth caused by preventable conditions such as PPH. The population for this current study consisted of midwives with one-year of experience working at primary health care (PHC) facilities rendering maternal health care services.

A non-probability, the heterogeneous purposive sampling method was used to sample participants who had the same characteristics desired to meet the objectives of the study from fourteen PHC facilities. These are midwives who are rendering maternal health care services and PHC facilities. Only midwives working at PHC facilities of the two selected districts of Limpopo province with one-year experience rendering maternal health care services were included in this study. The following midwives were excluded from these study: those who just qualified with less than one year in maternal health care settings but working at PHC level; those who are working at the hospitals and other districts in Limpopo province and those working in other provinces. An interview guide was constructed with one central question; “What are the challenges experienced in the management of women with PPH”, this question was followed by probing for more information on the management of women guided by the answers given by participants. The researcher conducted an in-depth face-to-face interview with the participants at their workplace, which lasted for 30–45 minutes depending on how participants expressed their management of women who experienced PPH.

Data were collected from March to June 2017. Data were collected until saturation was reached by the eighteenth (18) participants. Field notes and voice recorders were used during data collection with the permission of participants. Data analysis began during data collection were similar information grouped together. The researcher transcribed each participant's interview at the end of data collection and continue to analyse data using eight steps of Tesch's open coding methods. The researcher (TIR) read all the transcripts, grouped similar ideas together as codes, send to (MSM & LM) to rechecked data again in order to categories new codes and finally themes and sub-themes were constructed with the help of the independent coder who was in agreement with the analysed data.^11^

Measures to ensure trustworthiness were applied where the researcher remained in the field for a long-time during data collection until data were saturated. Transcripts of analysed data were - submitted to the independent coder who further analysed the collected data. Datwas presented in (table 2) as two themes and five sub-themes. To ensure transferability, the researchers provided methodological evidence that the research study's findings could be applicable to other contexts, situations, times, and populations.^12^.

**Table 2:** Themes and sub-themes indicating challenges faced by midwives in relation to the Management of PPH (n=18)

Theme	Sub-themes
2. Challenges experienced by midwives managing women with PPH	Difficulty experienced resulting in feelings of frustrations and confusion
	Adherence versus lack of adherence during the management of PPH
	Lack of time and shortage of human resource inhibits guidelines consultation
	Abrupt PPH after normal delivery
	Delayed versus prompt response by emergency services

Ethical clearance was obtained from the University of Venda (SHS/16/PBC/34/1910) and permission to conduct the study and was obtained from the Limpopo Provincial Department of Health (Ref: 4/2/2). District Executive managers granted the researchers permission to gain access and entry to PHC facilities at Vhembe and Mopani. Participants signed a consent form after all the procedures, the purpose of the study was explained to them. Information on withdrawal and refusal to participate was also explained. Confidentiality and anonymity were maintained by assigning a code to each participant.

## Results

Table 1 below denotes that, eighteen (18) participants from PHC facilities of the two selected districts participated in this study. The majority of participants were between the ages of 40–49 which indicated matured midwives with vast experiences of more than five years. Only one male participated in this study and the majority were females.

**Table 1:** Demographic profile of participants N=18

Variables	No. (%) of participants (n 18)
**Age**	
**20–29**	01(5.5)
**30–39**	01(5.5)
**40–49**	09(50)
**50–59**	05(27.9)
**>60**	02(11.1)
**Gender**	
**Male**	01(5.5)
**Female**	17(94.5)
**Years in current position**	
**1–3**	02(11.1)
**4–5**	02(11.1)
**>5**	14(77.8)
**Total**	18(100)
**Qualifications**	
**Diploma**	09(50)
**Degree**	07(38.9)
**Advanced midwifery and neonatal nursing** **science**	02(11.1)
**Total**	18(100)

Table 2 indicated the challenges experienced by midwives in the management of women with PPH. The results revealed that midwives experienced difficulty in the management of women with PPH. Some failed to follow the guidelines during the management of women who were experiencing PPH. There were limited time and shortage of human resources that hindered them from consulting the guidelines. When there was a need to transfer women who are experiencing PPH to the hospital, there was delayed response by ambulance services.

### Subtheme 1: Difficulty experienced resulting in feelings of frustrations and confusion

The results of the study showed that midwives had difficulty when managing women with PPH. Adhering to the maternity care guideline during the management of PPH was also difficult. However, they normally transferred women with PPH to the hospital and felt relieved after the women were transferred because they feared that the women might die in their hands. Midwives also indicated that they had to accompany the women to the hospital in their own cars because the ambulance was not available.

“*The woman started to bleed profusely after delivery. I changed the Ringers Lactate, inserted a new one with 20 units of Syntocinon added on it, rubbed up the fundus. I changed her linen and I repeated Syntocinon 10 units again. Hey, …it was difficult, and I felt unsure of what I was doing. I then called an emergency ambulance. I had to transfer and accompany the women using her own transport because the ambulance was delaying. I was relieved when I reached the hospital*. (Female, 60 years with 30 years in service)

Midwives also highlighted that they lack of confidence and frustrations when women develop PPH and they felt unprepared and confused. In addition, they had difficulty in managing women with PPH, this was influenced by a lack of knowledge on what to do though others indicated knowledge of measures to apply in the management of women with PPH. This was indicated in this way:

“*It becomes difficult because one tends to miss some of the steps in the management of the patient*.”(Female, 49 years with 20 years of experience)

Another midwife concurred and highlighted that

“*HEY…. It happened a long time ago, I cannot relate it well. I still remember one woman whom I met with PPH. I still remember injecting her with syntometrine, and though she was injected, the bleeding did not stop. Hmm…. It was confusing*, (Female, 47 years with 8 years of experience)

The same midwife went further and indicated a lack of knowledge as follows;

“*I rubbed the fundus and inserted the drip. I even wrap ice packs and put them on the abdomen for the uterus to contract*”. (Female, 47 years with 8 years of experience)

The other midwife indicated that (showing frustrations):

“*Hey… When you are in an emergency situation, even when 3 minutes pass, you fill that it is delaying. You know what, I was sweating a lot and afraid, not knowing what to do*”. (Female, 49 years with 5 years of experience)

Midwives reported barriers in the implementation of guidelines that affected the management of women with PPH. They further reported lack of honesty through women not providing an accurate history of PPH and delayed ambulance services were identified. A midwife verbalised that:

“*I managed a woman, who was gravida 4, she came while in labour and only attended ANC once. She did not give [me the] accurate history of [regarding] PPH. I progressed her well until she was fully dilated, the bladder was emptied. After delivery, I did everything [necessary] and even expelled the clots, but the patient was bleeding profusely. The women ended up [being] transferred to the hospital*”. (Female, 49 years with 5 years of experience)

### Subtheme 2: Adherence versus lack of adherence to maternity care guidelines during the management of PPH

Midwives reported that being the only midwife in the facility, especially at night contributed to the failure to follow and implement maternity care guideline. In most cases, midwives were working with junior nurses who followed midwives' orders. It was further highlighted that successful implementation needs at least two midwives who will remind and assist each other in case one forgets steps in the management of women. Midwives indicated that:

“*Because we are understaffed with limited sessions for in-service education, adherence to guidelines is difficult. It is easy to work being two midwives because you [can] advise each other on what to do. If I am alone and forget something, the assistant nurses will not remind me*”. (Female, 42 years with 18 years of experience)

Another midwife concurred:

“*There was no time to follow the guideline; I have to manage the patient through experience. Like with the patient we are talking about [referring to], I did not have time to open it [Maternity Care Guidelines], I just manage the patient*”. (Female, 51 years with 10 years of experience)

Another midwife also concurred and indicated that she had mastered the skills of managing women with PPH because their facility has experienced more cases of women with PPH.

“*It will have been easier to implement the [maternity care] guideline because one midwife will be checking the guideline while the other will be implementing its information on the patient, but I think I have mastered the skills of PPH management since this year, we had a lot of cases with PPH*”.(Female, 49 years with 7 years of experience)

One midwife reported differently, that:

“*But most of the time, I am used to implement the maternity care guideline. It is the one that helps me a lot*”. (Female, 47 years with 8 years of experience)

### Subtheme 3: Lack of time and shortage of human resource inhibit guidelines consultation

Midwives reported having experienced a shortage of resources and even a shortage of human resources. At PHC facilities one midwife was found managing all patients, including maternity cases. The shortage could result in the poor implementation of the guidelines, whereas, the time for in-service education and drills was not there. Participants indicated that management was done according to experience as there was no time to consult the guidelines. Midwives reported that:

“*There was no time to check what the guideline says, there was no one to help me, shortage of midwives is a problem in our facility, so I managed the women according to the knowledge that I have*”. (Female, 47 years with 5 years experience)

And:

“*It is not easy to manage the women alone as a midwife, practically, I was not using the guidelines, by then I just manage the women haphazardly because I know what to do and using the guidelines alone, in reality, is not possible*”. (Female, 51 years with 10 years experience)

In contrast, a midwife revealed that:

“*I am used to refresh [update] myself with the PPH guidelines when I am not busy. So that when I came across such a condition, I know what to do, because it is difficult to consult a guideline when one is having a real patient. Like the patient we are talking about, I did not have time to open it, I just manage the patient, it is not easy when one is alone with a junior nurse and there is no time to open a guideline*. (Female, 47 years with 8 years of experience)

### Subtheme 4: Abrupt PPH after normal delivery

Participants reported that some women fail to disclose their previous history of PPH during their first ANC visits hence midwives experienced the abrupt occurrence of PPH which was not anticipated. This was made worse by a lack of resources at the health facility. Women bleed without any signs even after the exploration of women's perineum by midwives. Thus, midwives must always be prepared to handle emergencies that all occur abruptly as whether there is a prior history of PPH or not the development of PPH may occur. Midwives shared their experience in this way that;

“*I did a follow-up and discovered that all her children were delivered at the hospital because of the same problem. She did not disclose this information during her first visit to the professional nurse who assisted her*”. (Female, 43 years with 15 years of experience)

“*The women delivered well, but immediately after delivery, the women started to bleed profusely with no reasons, the women were not having any visible tears in her perineum and vagina*”. (Female, 49 years with six years of experience; Female, 49 years with 5 years of experience)

Other midwives added

“*I was on call and delivered her, managed the third stage of labour. The woman was made comfortable, on monitoring she was found with clammy skin and low blood pressure. I quickly check the pads and found that the woman was soaked in the pool of blood*”.(Female, 60 years with 30 years' experience)

### Subtheme 5: Delayed versus prompt response by emergency services

The ambulance services were not complying with the norms and standards set by the department of arrival within sixty minutes to transfer women to the hospital. Midwives reported delay in ambulance services though few indicated prompt arrival. This was verbalized by midwives in this way:

“*The ambulance took more than one hour to collect the patient to the hospital*”. (Female 60 years with 30 years of experience)

“*The ambulance took 1 hour 30 minutes to 2 hours to collect the patient from here to the hospital*”. (Female 58 years with 24 years of experience)

And:

“*I then called an ambulance which took hours before arriving like about 3 hours. By then the woman was stable. I was relieved when the ambulance took her to the hospital*”. (Female, 49 years with six years of experience)

On the contrary, another midwife with a different version indicated that;

“*I then transferred her to the hospital; the ambulance did not take time because they are stationed nearer*”. (Male, 47 years with 17 years of experience)

## Discussion

Most participants in this current study were middle-aged with many years of experience. Having more years of experience would be an indication of a high level of expertise, though this depends on repeated exposure of midwives to PPH management. Majority of midwives who participated in the current study were females with only one male, this was influenced by the kind of profession which need a caring heart of women.^13^ Irrespective of that, some midwives experienced difficulty, frustrations, and confusion in the management of PPH. Though midwives indicated their management experiences, some lacked knowledge hence there was poor adherence to the maternity care guidelines during the management of women with PPH. The findings of this study were similar to other authors including a group of Association of Ontario for Midwives (AOM). The authors indicated the management of women with PPH as a stressful and difficult situation.^8^,^10^,^13^.^14^ Other midwives indicated that they communicated with God through prayers to give them wisdom and knowledge in the management especially when attending a woman alone.^8^ A study conducted on midwives in Ghana revealed that midwives had knowledge regarding the management of women with PPH, but their management skills were poor. Some midwives even failed to recognize women who were deteriorating and also failed to apply the learned knowledge.^15^, ^16^ Even during difficult situations, midwives need to stabilize the women through resuscitation by giving uterotonic drugs.

At PHC facilities, guidelines to manage PPH are kept, however, in-service education and Emergency Obstetric Simulation Training (EOST) drills were rarely conducted due to limited human resources. For the midwives to be able to practice EOST, equipment is needed and as such, they failed to do so because of lack of resources. Every consulting room must have its own equipment such as blood pressure machines, maternity care guidelines. Each facility must be provided with mannequins so that midwives can practice the learned information from various training hence improved skills. But as for now, the state of PHC facilities hinders midwives to do so because of the shortage and unavailability of equipment. The saving mothers report recommended that to improve the knowledge and skills of midwives in the management of PPH, EOST drills are to be conducted regularly.^4^ Being allocated as a midwife alone during the night and sometimes during the day had a negative effect in midwives adherence to maternal guidelines hence management of women with PPH was found to be poor. As such, other authors indicated that poor adherence to the guidelines was influenced by the stressful nature of the women's condition and insufficiency of staff where midwives failed to massage the women's uterus as indicated by the guideline and also delayed administration of Oxytocin.^17^, ^18^ Other midwives failed to adhere to the guideline because they were not aware of the information inside the guidelines. This was influenced by the way updates on guidelines were conducted as they did not cover all midwives. 17 Midwives reported that only a few midwives were updated on the maternal guidelines hence they did not report back to their colleagues hence they lacked information.

Midwives who trained a long time ago were not willing to implement updated information hence stick to old information learned during their basic training.^19^ Other midwives indicated their failure to adhere to the maternal guideline properly because the content inside the guideline was superficial, not clear and information was contradictory. Some of the midwives were not aware of the availability of such guidelines because they were not distributed to all facilities.^3^, ^19^ The implication of poor adherence to maternal guidelines might lead to increased maternal morbidity and mortality rate hence failure to reach Sustainable Developmental goals by 2030.^20^ It was recommended that continuous education, regular drills and adherence to guidelines by midwives are vital in reducing maternal mortality rate caused by PPH.^8^ Various authors discovered that continued training had small but significant improvement in the knowledge and quality of care rendered and as such to be done regularly in order to reinforce adherence of guidelines by midwives.^17^, ^22^

The shortage of staff resulted in a limited time of taking history even during subsequent visits. Midwives are always in a hurry to can see all the patient who is on the queue daily, hence they tend to miss other important information that is crucial during pregnancy and as such, they are mostly surprised by an abrupt PPH even in women whom they did not anticipate its occurrence. As such, midwives must always be ready because PPH might happen to every woman regardless of the history of previous PPH. Knowledge, skills, and availability of resources will determine the readiness of midwives and should always take history among women. 21, 24, 25, 26 24 Failure to take an accurate history and shortage of staff was also indicated in a study conducted on midwives in Ghana where midwives delegated junior nurses on other duties regarding Active Management of the Third Stage of Labour (AMTSL) as a means of preventing PPH.^23^ For effective management of PPH, skilful clinicians must attend to the women who are experiencing PPH with the required resources for management.

The woman who is experiencing PPH is in an emergency state that requires quick action because she might collapse within a short period of time, hence more than one competent midwife who must act fast in initiating the management must attend to her. The condition needs to be managed systematically following steps.^8^,^27^ This means that more than one midwife is needed as indicated by participants, one will be checking the steps either in the guideline or protocols hence another one will be implementing them. Various studies indicated difficulties experienced by a midwife in managing a pregnant woman alone hence, they recommended that more than one midwife must be involved in the management though the shortage of staff makes this difficult.^5^, ^9^, ^10^ All women who experienced PPH after delivery must be transferred to the hospital for haemoglobin assessment and management as indicated by the maternity care guidelines of South Africa.^5^

The researchers discovered that some midwives were frustrated by the ambulance services that took longer than required to can transfer the women who needed further treatment. This was especially experienced by women who continued to bleed after all the management known rendered by midwives.^28^ For the ambulance services must arrive within sixty minutes to collect the women to the next level.^29^ Delays are mainly due to poor communications, transport shortage, long-distance and poor roads.^17^,^30^, ^31^,^32^ The implications of ambulance delays have led to delay in initiation of treatment by the next level hence women's conditions deteriorate increasing the rate of maternal deaths.

## Conclusion

The researcher discovered that midwives were faced with various challenges and difficulties in the management of women with PPH though some knew the management others did not know the measures used in the case of PPH. Midwives were frustrated because of the shortage of human resources, where one midwife was allocated alone during the night and others during the day. Delay in ambulance services added to their frustrations due to fear that the women might die in their care. Women did not disclose their previous history of PPH and failed to co-operate when midwives advise them to do so, hence, midwives experienced a sudden gush of blood from women that they did not anticipate. All this contributed to poor implementation of the guideline hence an increase in the number of maternal mortality rates.

## Limitations

The study was limited to the selected districts of Limpopo province and can not be generalised to other district and provinces in South Africa given the purposive nature of midwives sampling.

## Recommendations

Midwives must update themselves on matters pertaining to maternal health care services through personal development in order to be acquainted with the information contained inside the guidelines. To follow the guidelines during the management of PPH women in order to reduce maternal morbidity and mortality rates. To work with home-based careers and the clinic committee on educating women about the importance of giving correct information during history taking. To reinforce education by advising women to mention their previous history of PPH every time the women visit the clinic for antenatal care. To enhance midwives' competencies; The Department of Health to purchase teaching model/manikin and equipment's needed by midwives and be distributed to each facility for midwives to practice EOSOT drills which included management of women with PPH; Offer continuous development and in-service training in order to keep midwives up to date; to increase the uptake of midwives for the purpose of advance midwifery training so that midwives acquire advanced skills in managing obstetric emergency conditions hence improve good maternal outcomes; to purchase more ambulances designated for maternal health care services only. Facility managers to improve maternal health care services must do auditing of maternal records of women managed by midwives with PPH. Further research needs to be conducted on perceptions of managers towards guidelines implementations by midwives as their support to midwives when implementing maternal health care guidelines is vital in the reduction of MMR.
